# Evaluating Cross-Linking Efficiency and Cytocompatibility of Three Commonly Used Photoinitiators across Different Cell-Compatible Hydrogel Platforms

**DOI:** 10.1021/acs.biomac.5c01142

**Published:** 2025-09-22

**Authors:** Aya Gavish Moscovitz, Haneen Simaan Yameen, Orit Bar-Am, Dror Seliktar

**Affiliations:** † The Faculty of Biomedical Engineering, 108811 Technion-Israel Institute of Technology, Haifa 3200003, Israel; ‡ The Interdisciplinary Program in Biotechnology, Technion−Israel Institute of Technology, Haifa 32000, Israel

## Abstract

Biomedical hydrogels often use a photopolymerization strategy to cross-link the polymer network. There are only a few cyto-compatible photoinitiators (PIs) that are commonly used for cross-linking biomedical hydrogels, including Irgacure 2959, lithium phenyl-2,4,6-trimethylbenzoylphosphinate (LAP), and Eosin Y. Herein, we tested these PIs to optimize the cross-linking efficiency while minimizing cell death. Testing was performed on three types of hydrogels, including a synthetic material (poly­(ethylene glycol)-diacrylate, PEG-DA), a semisynthetic material, PEG-fibrinogen (PF), and a modified biological material, methacrylated fibrinogen (FibMA). The results showed that PI concentration and illumination intensity had a significant impact on cross-linking efficiency, as measured by the shear storage modulus, with each material demonstrating different responses to the photopolymerization parameters. Optimal photo-cross-linking conditions were not the same for the modified protein hydrogels as compared to synthetic and semisynthetic materials. These findings may have consequential implications when applying photopolymerization to cross-link various types of cell-compatible hydrogels for biomedical applications.

## Graphical abstract

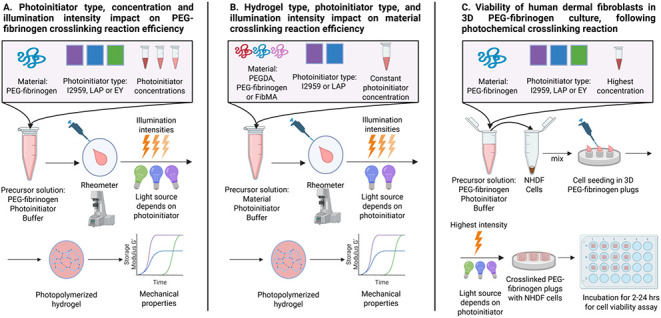

## Introduction

Hydrogels are viscoelastic polymeric materials that are organized into three-dimensional (3D) networks containing large amounts of water. The polymeric networks are composed of physically and/or chemically cross-linked hydrophilic polymer chains, which can be cast into many shapes and sizes according to the specified requirements.^1,2^ Hydrogels can be used for many applications in medicine due to their biocompatibility and similarity to natural soft tissue.^3^ For example, these materials have been used as wound dressings or intraocular lenses with excellent clinical outcomes.^4,5^ Cell-compatible hydrogels are a subclass of materials made specifically for applications that require cells to grow within them. Applications in cell therapy and tissue engineering are examples of where cell-compatible hydrogels are routinely used.^6^ These applications necessitate more stringent physical and chemical specifications premised on biomimicry of the extracellular matrix (ECM).^1,7^ Other important characteristics of cell-compatible hydrogels are their ability to undergo cyto-compatible gelation (i.e., in the presence of cells) and controlled degradation, preferably by cell-mediated pathways (i.e., protease-mediated degradation).

Most hydrogels are made from either synthetic or natural materials.^8^ For example, biological adhesives or sealants for wound healing and surgical procedures are made from reconstituted fibrin or gelatin.^9^ Synthetic hydrogels are constructed from synthetic polymers such as poly­(ethylene oxide) (PEO) or poly­(vinyl alcohol) (PVA).^10^ Some surgical sealants are made from chemically modified synthetic materials that enable controlled gelation, such as diacrylate-modified poly­(ethylene glycol) (PEG) or PEG-DA.^4^ Synthetic hydrogels used in drug delivery systems are also made from modified PEG, namely, PEG-dimethacrylate (PEG-DMA).^5^ There are also hydrogel materials that are semisynthetic or hybrid formulations, where peptides, proteins, or polysaccharides are conjugated to synthetic hydrophilic polymers to form adducts.^2,11^ Semisynthetic hydrogels made from these adducts are designed to be structurally stable, mechanically versatile, biocompatible, and biodegradable.^1^

PEGylated protein hydrogels are an example of a semisynthetic design,^12^ where PEGylation is the process of PEG conjugation to a protein via covalent chemistry.^13–16^ PEGylated protein hydrogels leverage the semisynthetic paradigm to endow the protein materials with additional strength or chemical versatility;^8^ the PEG is used to control the mechanical and physical properties of the material, and the protein provides cell adhesion, biodegradation, and other cues for tissue generation.^2,12^ PEG-fibrinogen (PF) hydrogels are an example of this design, which our group uses for tissue engineering, cell delivery, and controlled drug release applications.^13,14,17,18^

Methacrylated natural macromolecular hydrogels are another example of chemical modifications that are made to natural hydrogels for more versatile use in biomedical applications.^19^ They comprise modified natural polymers such as hyaluronic acid (HA), gelatin, or fibrinogen, which are chemically modified with methacryloyl (MA) groups to form HA-MA,^20^ GelMA,^21,22^ or FibMA,^23^ respectively. The formation of a hydrogel is accomplished by a mild chemical cross-linking between the acrylate groups that must maintain high cell viability.^24^ These chemically modified hydrogels preserve the biological advantages of bioactivity and biodegradability. They can also enable more controllable cross-linking reactions through these chemical modifications, leading to improved mechanical properties.^25^ They have been used extensively for tissue engineering, cell therapy, and drug delivery applications.

Most chemically modified natural macromolecules, semisynthetic, and synthetic materials can be cross-linked into a hydrogel by either chemical or physical reactions, with the sol–gel transition occurring upon external stimuli such as changes in pH, temperature, light, ultrasound, or other conditions.^2,8,26^ Light-activated polymerization (i.e., photopolymerization) is a common technique to chemically cross-link cell-compatible hydrogels.^27–29^ The cross-linking mechanism is based on a free-radical chain propagation reaction, utilizing a photoinitiator (PI) and a corresponding light source to create free radicals that initiate a polymerization reaction.^30–33^ Photopolymerization has several advantages, including the ability to perform the reaction in an aqueous medium. It is a very fast process that takes from a few seconds to minutes, which is advantageous for in situ hydrogel formation. It also allows for excellent spatial and temporal control of the cross-linking process.^34^ Photopolymerization at ambient or body temperature and under physiological pH levels has been documented to have either minimal or moderate effects on cytotoxicity and cell viability,^30,35,36^ depending on the type of hydrogel, the PI, the light source, and the cell type used. The cytotoxicity of photopolymerization becomes acutely critical when applied to cell-laden hydrogels.^24,29,37–40^

Among the factors that can potentially reduce cytotoxicity of photopolymerization is the choice of PI. There are several PIs used for hydrogel photopolymerization that are suitably mild in terms of radical formation to be used in the presence of cells and tissues but are sufficiently water-soluble and reactive enough for facilitating hydrogel cross-linking. The three most prevalent PIs in this context are 2-hydroxy-4′-(2-hydroxyethoxy)-2-methylpropiophenone (Irgacure©2959, I2959); lithium phenyl-2,4,6-trimethylbenzoylphosphinate (LAP), and Eosin Y disodium salt (EY).^30,35^ PIs are generally divided into two mechanistic classes: Type I (cleavage-type) and Type II (electron-transfer-type), each with distinct implications for use in biomedicine. Type I photoinitiators, such as I2959 and LAP, generate radicals through direct photolytic cleavage of a labile bond upon light exposure, forming two highly reactive radical species capable of initiating polymerization without requiring additional co-initiators^41,42^ (Figure 1A,B). In contrast, Type II PIs like EY operate via a photoinduced electron transfer (PET) mechanism that requires a co-initiator, commonly an amine such as triethanolamine (TEA). Upon excitation by light, EY accepts an electron from TEA, generating a radical anion (EY^•–^) and a radical cation (TEA^•+^); the latter undergoes α-cleavage to yield the initiating radical^30^ (Figure 1A). These differences affect the reaction kinetics and cytocompatibility of the radical polymerization process. As such, cross-linking reactions are not consistent across these different PIs and can also be affected by the hydrogel precursors, including the monomer concentration, degree of functionalization, reactivity of functional groups, and the curing depth of the sample.

**Figure 1:**
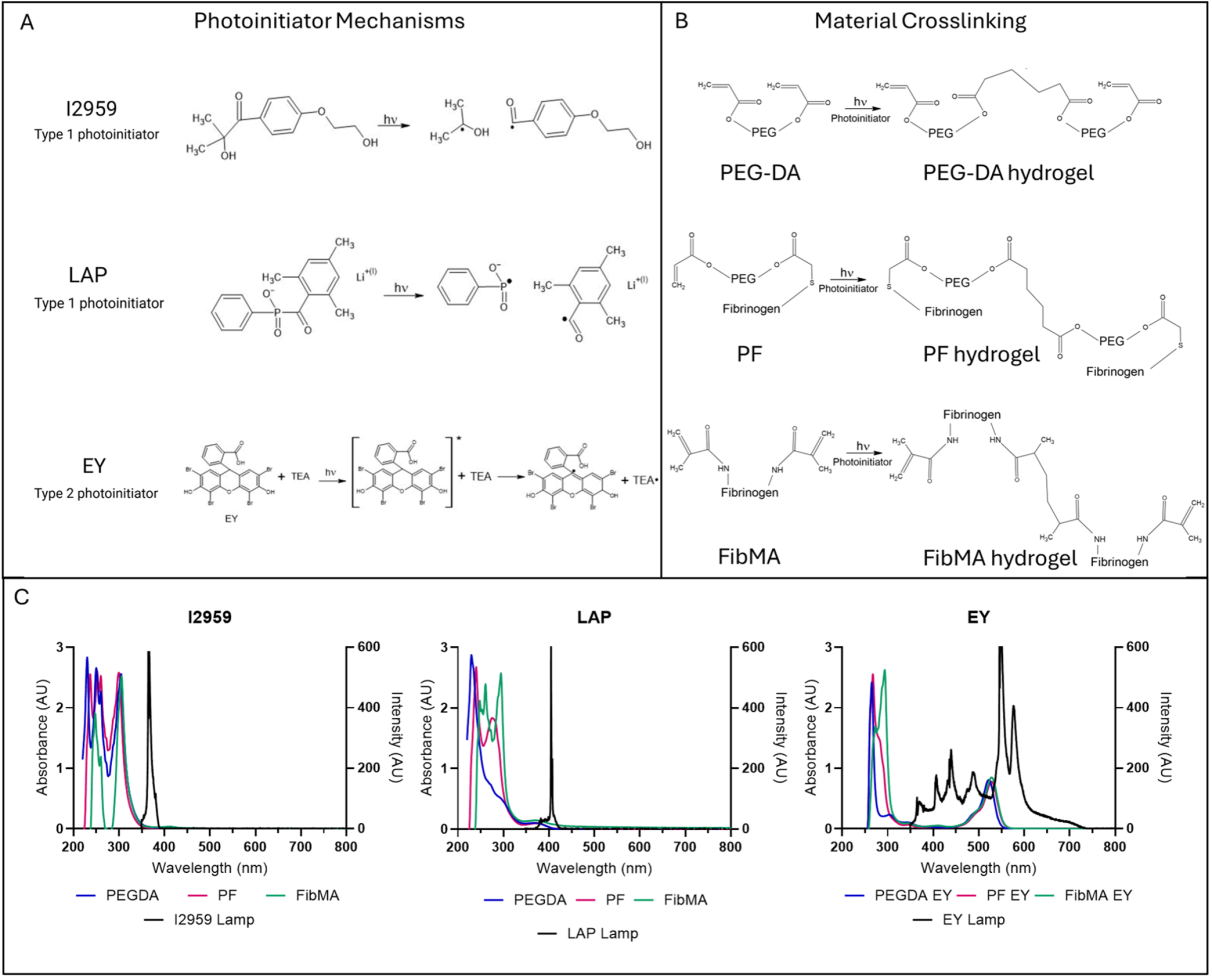
Photoinitiators are commonly used for making cell-compatible biomedical hydrogels. (A) The mechanism of photoinitiation for Type 1 and Type 2 PIs used in the present study. (B) Radial photopolymerization of three different types of hydrogels used in the present study. (C) Absorbance spectra of each PI in the respective hydrogel precursor solution, superimposed with the light intensity spectra of the respective light source used for the photoinitiation (all light sources include filters, as indicated in the Materials and Methods section).

Although there are many publications that describe photopolymerization as a function of reaction parameters,^43^ including light intensity and concentration of reactive groups and photoinitiators, these investigations are typically not carried out on biomedical hydrogels.^44^ Indeed, the predictive models of radical polymerization photochemistry that have been reported in the literature using traditional polymer materials^45^ do not apply to cell-encapsulating biomedical hydrogels made from dilute solutions of proteins or polysaccharides, where different PIs and light intensities are used to ensure cytocompatibility.^20,29,37,40,46^ Moreover, nearly all of the conventional polymer systems with which existing models of photopolymerization kinetics have been studied are not amenable to cell encapsulation, limiting any possible correlation between photopolymerization kinetics and cell viability in these prior studies.

For these reasons, it is important to study photochemistry kinetics and cytotoxicity using encapsulating biomedical hydrogels to understand how the choice of PI and cross-linking conditions (e.g., PI concentration, irradiation intensity) of a biomedical hydrogel can not only impact the viability of encapsulated cells but also affect the characteristics of the final hydrogel product, including mechanical and diffusional properties, uniformity, homogeneity, biodegradability, and biofunctionality.^30^ In the present study, we set out to better understand how PI type, concentration, and irradiation intensity affect one of these important characteristics, the cross-linking density (i.e., material modulus), using different types of encapsulating biomedical hydrogels that are suitable for 3D cell growth. The hydrogels include a modified protein-based material (FibMA), a hybrid material (PEG-Fibrinogen), and a synthetic material (PEG-DA). We compare the effects of two different PIs, I2959 and LAP, on the three hydrogel systems. We also evaluated photo-cross-linking of the PF hydrogels using I2959, LAP, and EY to compare cross-linking efficiency with three different commonly used PIs. We evaluated the PI concentration and irradiation intensities at the PIs’ recommended excitation wavelength (365, 405, and 525 nm for I2959, LAP, and EY, respectively). We report optimal cross-linking conditions for each PI in terms of PF hydrogel mechanical properties using rheological measurement, namely, the maximum shear storage modulus, *G*′, as well as the cross-linking kinetics. We further reported the viability of cells encapsulated within the PF hydrogels to verify cytocompatibility of each respective PI under the optimal cross-linking conditions. We specifically examined cytocompatibility with neonatal human dermal fibroblasts (NHDFs), which are known to be relatively tolerant to photochemical cross-linking.

## Materials and Methods

### Preparation of Hydrogels

#### PEG-DA Preparation

PEG-diacrylate (PEG-DA) was synthesized from 10 kDa PEG–OH (Merck, Kenilworth, NJ) as described elsewhere.^13^ Briefly, PEG–OH acrylation is carried out under argon by reacting a dichloromethane (DCM, Aldrich, Sleeze, Germany) solution of the PEG–OH with acryloyl chloride (Merck, Darmstadt, Germany) and triethylamine (Fluka) at a molar ratio of 1:1.5:1.5 (0.2 g of PEG/mL of DCM). The final product is precipitated in ice-cold diethyl ether and dried under vacuum overnight. The degree of end group conversion of 97–99% was confirmed by ^1^H NMR. PEG-DA hydrogel precursor solution was prepared by dissolving PEG-DA to 5% w/v (50 mg/mL) with phosphate-buffered saline (PBS)^37^ and adding a photoinitiator at the desired concentration. The hydrogel formation was accomplished by photopolymerization of the PEG-DA precursor solution under various conditions of photoinitiators and irradiation intensities, as described in the Results section.

#### PEG-Fibrinogen (PF) Preparation

For the PF synthesis, conjugation of the linear PEG-DA to thiol groups on denatured fibrinogen (Human fibrinogen: Sigma-Aldrich or Tisseel, Baxter; Bovine-fibrinogen: ID-bio or Bovagen) was done according to a PEGylation protocol as described elsewhere.^13,47^ Briefly, tris­(2-carboxyethyl) phosphine hydrochloride (TCEP-HCl, Sigma-Aldrich, St. Louis, MO) was added to a 7 mg/mL solution of fibrinogen in 150 mM phosphate-buffered saline (PBS) with 8 M urea (molar ratio of 1.5:1 TCEP to fibrinogen cysteines). Linear 10 kDa PEG-DA was reacted for 3 h with the protein at a 4:1 molar ratio of PEG to fibrinogen cysteines. The PF conjugate was precipitated in acetone and redissolved in PBS containing 8 M urea to a 10–12 mg/mL final protein concentration. The PF conjugate was then dialyzed against PBS at 4 °C for 1 day (Spectrum, 12–14 kDa MW cutoff, California), sterilized, and characterized according to previously published protocols.^47^ The protein concentration was determined using a BCA Protein Assay kit (Pierce Biotechnology, Inc., Rockford, IL). To establish the total PEG-protein concentration, 0.5 mL of the PF precursor solution was lyophilized overnight, and the mixture was weighed. The amounts of the total PEGylated product (dry weight) and protein content (BCA result) were used to calculate the PEG concentration in the precursor solution.^14^ The PF hydrogel precursor solution was prepared by diluting the PF solution to 8 mg/mL protein with PBS^37^ and adding a photoinitiator at the desired concentration. The hydrogel formation was accomplished by photopolymerization of the PF precursor solution under various conditions of photoinitiators, concentrations, and irradiation intensities, as described in the Results section.

#### Methacrylated Fibrinogen (FibMA) Preparation

FibMA was synthesized with a 20% molar ratio of methacrylic groups chemically attached to the free amines of fibrinogen molecules (i.e., FibMA_0.2_) as described elsewhere.^23^ A solution of 1% w/v fibrinogen was prepared using 150 mM PBS with 8 M urea. The solution pH was adjusted to 9.4 with 2 M NaOH. Methacrylic anhydride (0.8% v/v, Sigma-Aldrich, Buchs, Switzerland) was then added to the fibrinogen solution while maintaining the pH between 9 and 9.4. The mixture was incubated for 3 h in the dark at room temperature under stirring. Subsequently, the solution pH was adjusted to 7.4 with 2 M HCl. The final product was purified using an ultrafiltration system with a 50 kDa cutoff membrane (PALL Life Sciences, New York) against purified water. The purified FibMA was then mixed with an excipient sugar, either sucrose or trehalose, in solution at a 1:1 mass ratio (sugar to protein), aseptically filtered through a 0.22 μm filter (Merck KGaA, Darmstadt, Germany), and lyophilized. The sucrose/trehalose is an excipient often used to help prevent irreversible protein–protein interactions during freezing and after lyophilization, which can lead to solubility issues. We confirmed that sucrose/trehalose at the specified concentrations did not affect the cross-linking reaction using rheological testing. The lyophilized FibMA was stored at −80 °C and used within 6 months. FibMA hydrogel precursor solution was prepared by dissolving lyophilized FibMA in a 25 mM HEPES buffer solution (Sartorius, Biological Industries, Beit Haemek, Israel) to a concentration of 40 mg/mL and adding a photoinitiator in the desired concentration. HEPES was used because FibMA solubility was better in this buffer as compared to PBS. The hydrogel formation was accomplished by photopolymerization of the FibMA precursor solution under various conditions of photoinitiators and irradiation intensities, as described in the Results section.

### Photoinitiators

Three different photoinitiator systems were used in this study. 2-Hydroxy-4′-(2-hydroxyethoxy)-2-methylpropiophenone (IRGACURE 2959, I2959; Ciba, Basel, Switzerland) stock solution was prepared with 70% ethanol. Cross-linking was conducted under ultraviolet (UV) light of 365 nm (OmniCure SERIES 1000, EXFO, with a mercury lamp, a high pass filter >350 nm, and a low pass filter <380 nm). Lithium phenyl-2,4,6-trimethylbenzoylphosphinate (LAP, Sigma-Aldrich, St. Louis, MO) stock solution was prepared with PBS. Cross-linking was conducted under 405 nm (X-Cite SERIES 120Q, EXFO, with a mercury lamp and a band-pass filter of 400–410 nm). Eosin Y disodium salt (EY) (Sigma-Aldrich, St. Louis, MO) stock solution was prepared with PBS. Cross-linking was conducted under visible light (Illuminoss Photodynamic Curing System, with a mercury lamp and a high pass filter >350 nm). EY requires two cofactors to initiate the polymerization reaction (at a constant percentage): 0.39%v/v 1-vinyl-2-pyrrolidinone (NVP, Sigma-Aldrich, St. Louis, MO) as a comonomer and 1.5%v/v triethanoleamine (TEOA, Merck, Darmstadt, Germany) as a co-initiator.^48–50^

### Rheological Measurements

The hydrogel rheological measurements of gelation, mechanical properties, and cross-linking kinetics were carried out using a TA Instruments AR-G2 rheometer (New Castle, DE) equipped with a parallel plate geometry (diameter 20 mm). Each measurement was carried out with a hydrogel precursor solution containing one of the PIs: I2959, LAP, or EY, in different concentrations. The instrument was set to a gap size of 550 micrometers with approximately 170 microliters of precursor solution placed in the gap. The in situ polymerization of the samples was conducted by either UV light of 365 nm for I2959, 405 nm light for LAP, or visible light for EY. Time-sweep oscillatory tests were performed at 25 °C at a sinusoidal 2% strain rate and a 1 Hz angular frequency. A chamber was placed around the samples to minimize evaporation of the sample during testing.

### Cell viability

#### Cell-Laden PF Hydrogel Plug Preparation

Neonatal human dermal fibroblasts (NHDF) were thawed and seeded on tissue culture plastic (TCP) with growth medium containing Dulbecco’s modified Eagle medium (DMEM, Gibco, U.K.), 10% fetal bovine serum (FBS), and 1% penicillin–streptomycin–ampicillin (Biological Industries, Israel). The passaged cells (passage 9–12) were trypsinized using 0.25% trypsin-EDTA (Biological Industries, Israel), centrifuged at 200g for 10 min, and suspended with the PF hydrogel precursor solution, containing various amounts of PI. Hydrogel constructs (3D) were made from a volume of 75 μL of PF precursor cell-laden solution with 3 × 10^6^ cells/mL and placed in a 5 mm internal diameter cylindrical tube glued to a transparent glass slide. The constructs were photopolymerized for 3 min at 25 °C under the corresponding light source for each PI, to form a cylindrical plug construct with approximate dimensions of 5 mm diameter and 4 mm height. These 3D plugs were incubated in a tissue culture plate with the growth medium for additional analysis.

#### Qualitative Cell Viability

The viability of the NHDF cells encapsulated in hydrogel plugs was analyzed by a Calcein/Ethidium Live/Dead assay. Briefly, each plug was transferred to a new 24-well plate (one plug per well), containing a solution of 1 mL of PBS with 4 mM Calcein acetoxymethyl ester (green stain for live cells) and 2 mM ethidium homodimer-1 (red nuclear stain for dead cells, Sigma-Aldrich, Buchs, Switzerland), and incubated for 45 min on an orbital shaker at 37 °C. After staining, the plugs were washed with PBS for 5 min. Cells were microscopically imaged using a Zeiss LSM 700 confocal microscope (Carl Zeiss, Oberkochen, Germany).

#### Quantitative Viability

Quantitative viability assessments were made on the encapsulated NHDF cells (3 × 10^6^ cells/ml) within the PF hydrogel plugs made using the three different PIs. The hydrogel cylinder plugs (75 μL each) were photopolymerized by illuminating for 3 min with the respective light source that corresponded to the PI being tested as follows: I2959 (0.5% w/v) was illuminated at 5 mW/cm^2^, LAP (0.1% w/v) was illuminated at 4 mW/cm^2^, and EY (0.5 mM) was illuminated at 240 mW/cm^2^. The hydrogels were then placed in a 24-well plate with culture medium and incubated for up to 24 h at 37 °C and 5% CO_2_. The cells were harvested after 2 and 24 h. The PF hydrogel plugs containing cells were digested using collagenase, and the cells were harvested and stained with Trypan blue. Next, the viable cells were counted and compared to the total number of cells in the constructs at the two time points. The PF digestion process was achieved using 0.5 mg/mL Collagenase type 1A solution (Sigma-Aldrich, Steinheim, Germany) for 1.5 h at 37 °C with mild shaking, followed by centrifugation, redissolving in PBS, and staining with Trypan Blue as described elsewhere in more detail. Quantitative cell viability was investigated by counting the stained cells with an automated cell counter (Countess-Invitrogen).

### Statistics

Statistical analysis was performed using GraphPad Prism (GraphPad Software, San Diego, California). Data from at least three independent experiments, performed in triplicate, were quantified and analyzed for each variable. Comparison between multiple treatments was made with analysis of variance (ANOVA) using multiple comparison tests, and all data were presented with standard deviation (SD). A *p*-value of below 0.05 was considered statistically significant (marked with asterisks). Pearson correlation coefficients (*r*) between parameters were calculated assuming data were sampled from a Gaussian distribution.

## Results

### Photopolymerization Effects on the Mechanical Properties of Semisynthetic Hydrogels

The effects of PI type, PI concentration, and illumination intensity on hydrogel mechanical properties were comprehensively evaluated on a semisynthetic PF hydrogel using shear modulus measurements (depicted in Figure 2A). The PF hydrogel was prepared with each respective PI and characterized in situ during photopolymerization. For comparison with previously published studies, we used the conventional nomenclature for each respective PI (e.g., the concentration of I2959 and LAP PIs was reported in % (w/v), whereas EY concentrations are reported in mM).^30,35^ For comparison, a conversion table with all the PI concentrations in both % (w/v) and mM is provided in the Supporting Information. The shear storage modulus (*G*′) was measured up to the maximum cross-linking of the PF hydrogel with its respective PI, and the highest value was defined as the plateau *G*′. The shear loss modulus (*G*″) was also recorded but was not presented in the results. Importantly, the values of *G*″ were approximately 2 orders of magnitude lower than *G*′ for all the samples (not shown), indicating that full gelation occurred.^37^ The optimal parameter of each variable was identified using the maximum plateau *G*′ values, within the tested range. For example, the optimal PI concentration for each respective hydrogel system was determined by measuring the modulus of hydrogels made with different concentrations of PI, keeping all other parameters constant. The optimal PI concentration produced the highest cross-linking density (i.e., highest plateau *G*′, *G*′_plateau_). A similar strategy was used to determine the optimal light intensity. Finally, comparisons across the different hydrogel platforms were possible based on the relative optimization of *G*′_plateau_ and under the assumption that optimal conditions led to maximum cross-linking density of each respective hydrogel network.

**Figure 2:**
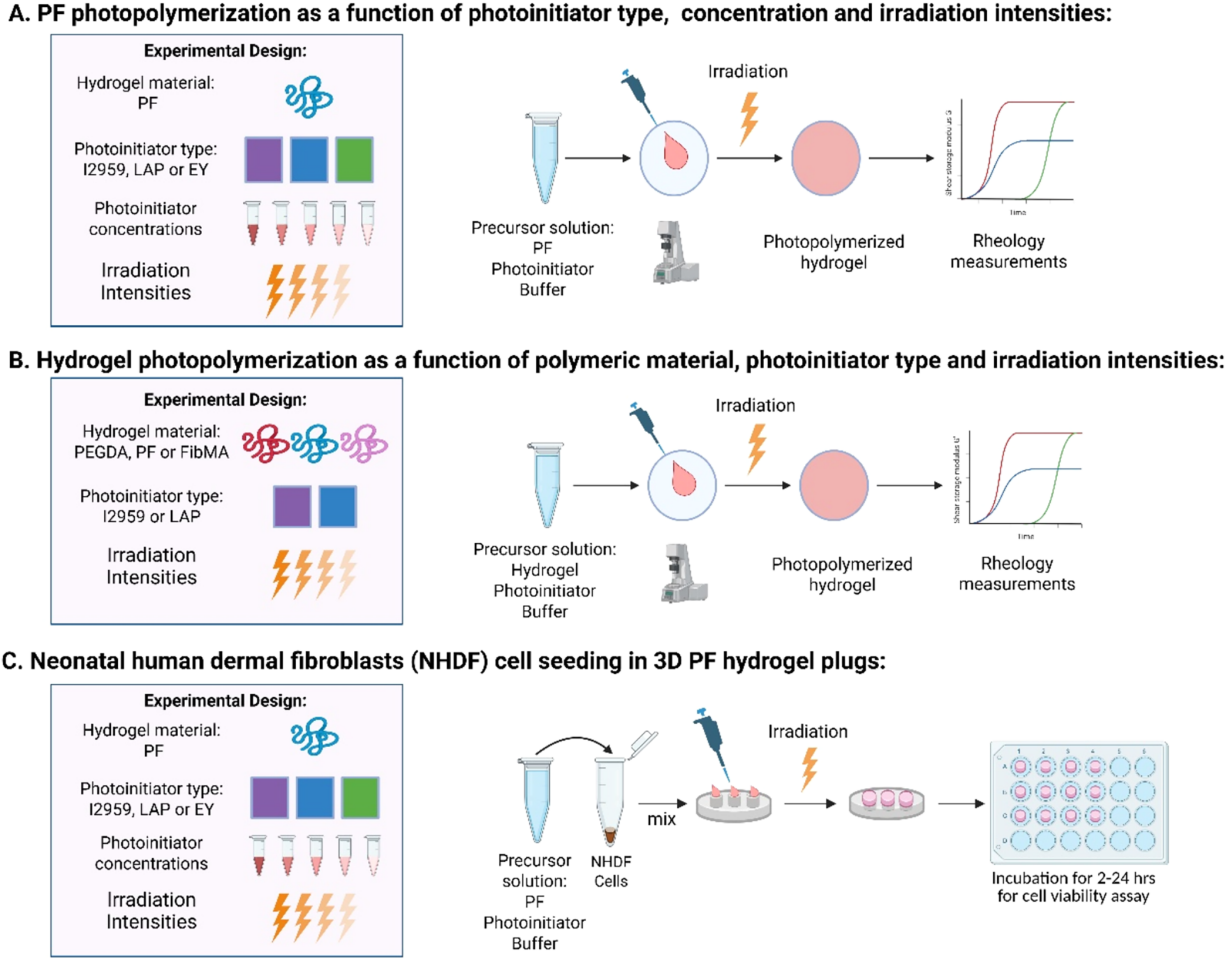
Experimental setup for testing the mechanical properties and cytocompatibility of photopolymerization with different photoinitiators and using various hydrogel types. (A) A semi-synthetic hydrogel made from PEG-fibrinogen (PF) was photopolymerized with three different PIs, at different concentrations and with different irradiation intensities. (B) Three hydrogel types were photopolymerized with two different PIs at different irradiation intensities. (C) Cytocompatibility was tested on neonatal human dermal fibroblasts following photopolymerization within PF hydrogels made using three different PIs.

#### Comparing Irradiation Intensities

The time-sweep *G*′ values of PF hydrogels under different irradiation intensities during photopolymerization are shown in Figure 3. Specifically, the increasing irradiation intensities, with constant PI concentration, are shown in Figure 3A–C for formulations containing I2959 (red), LAP (blue), and EY (green), at concentrations of 0.1%, 0.1%, or 0.1 mM, respectively. The corresponding *G*′_plateau_ values, determined from the time-sweep data, are presented in Figure 3D–F for the respective PIs. Each PI was tested at four or five different intensities within a range that were previously reported in the literature and/or that were empirically determined to reach a *G*′ maxima. Statistical analysis was performed between the *G*′_plateau_ data for the different intensities of each PI, and the *p*-values are indicated accordingly in Figure 3D–F. The PF precursor with 0.1% w/v I2959 (Figure 3A, red), illuminated at 365 nm with an intensity of 0.2–5 mW/cm^2^, resulted in noticeably higher levels of *G*′_plateau_ as compared to PF with 0.1% w/v LAP (Figure 3B, blue), illuminated at 405 nm with an intensity of 0.1–4 mW/cm^2^. PF with EY 0.1 mM (Figure 3C, green), illuminated with visible light between 30 and 240 mW/cm^2^, was similar to I2959 in terms of *G*′ values. Notably, I2959 and EY reached similar *G*′_plateau_ values of around *G*′ = 280 Pa for the fully cross-linked hydrogel (at 2 and 240 mW/cm^2^, respectively), while LAP *G*′_plateau_ value peaked at about 200 Pa, almost 30% less (with 0.2 mW/cm^2^). I2959 and LAP demonstrated a peak of optimal irradiation intensity to achieve this *G*′_plateau_, whereas EY did not reach optimal conditions and could not be tested at higher intensities due to limitations with the light source. A two-way ANOVA confirmed significant differences in the maximum *G*′_plateau_ of the hydrogel as a result of changing the intensity and the PI type (*n* ≥ 6, *p* < 0.005). Another notable observation from these data is the higher cross-linking kinetics with increasing intensity for all PI types.

**Figure 3:**
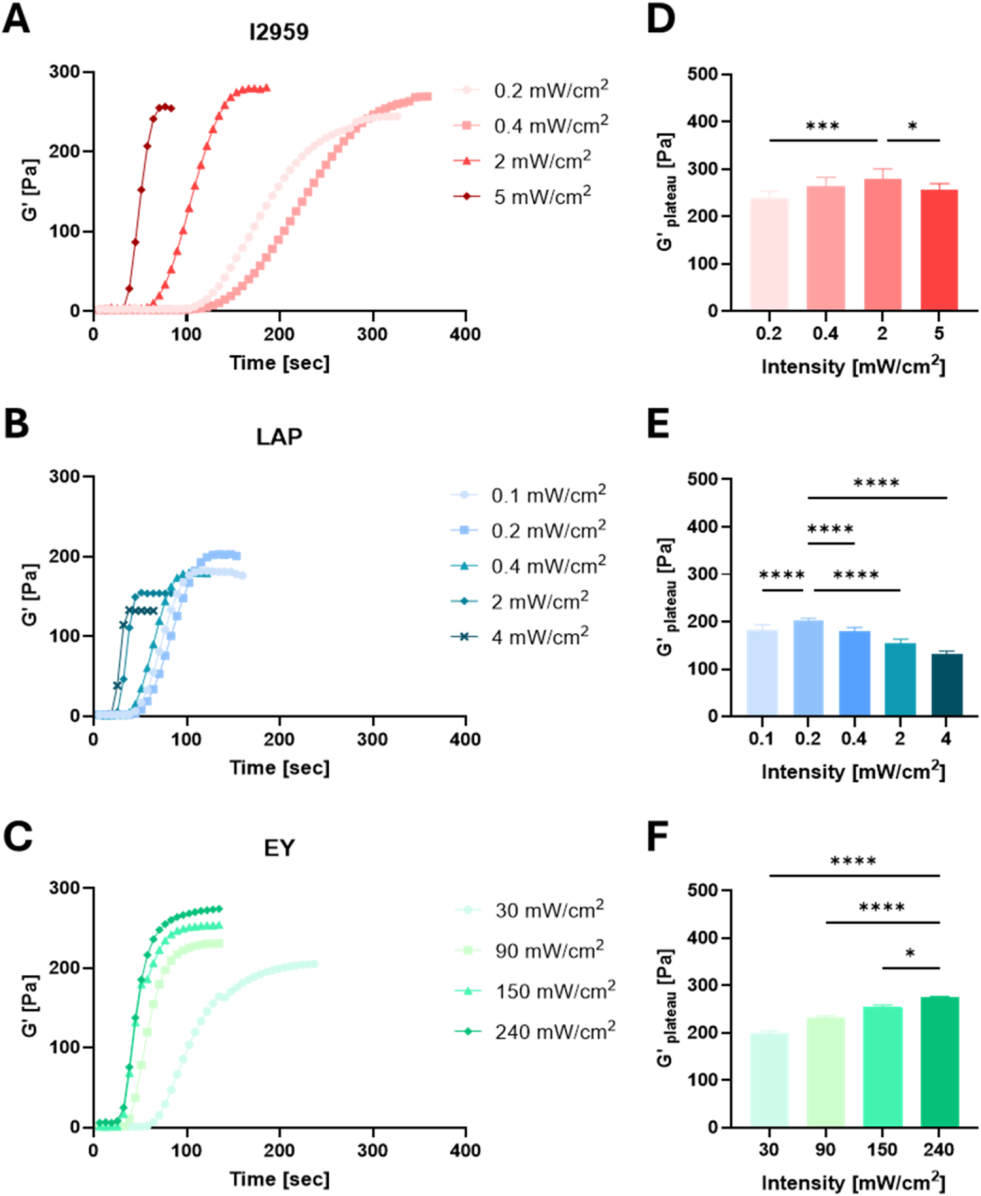
Effect of light intensity on shear storage modulus (*G*′). Time-sweep measurements (A–C) of PF hydrogels (8 mg/mL) that were photopolymerized in situ using a rheometer at room temperature under constant strain show increasing shear storage modulus, *G*′[Pa], with different light intensities; irradiation was initiated after 12–15 s. (A, C) I2959 at 0.1% w/v, 365 nm, 0.2–5 mW/cm^2^; (B, E) LAP at 0.1% w/v, 405 nm, 0.1–4 mW/cm^2^; (C, F) EY at 0.1 mM, visible light, 30–240 mW/cm^2^. The maximum *G*′_plateau_[Pa] was reported for the different PIs and light intensities (*n* ≥ 6). Statistics were calculated using one-way ANOVA—Dunnett’s multiple comparisons test; results are presented in comparison with the largest value column (SD, *P < 0.05, **P < 0.01, ***P < 0.001, and ****P < 0.0001).

#### Comparing PI Concentrations

A similar analysis comparing the *G*′ of PF hydrogels during photopolymerization with increasing PI concentrations is shown in Figure 4A–C. The corresponding *G*′_plateau_ values, determined from the time-sweep data, are presented in Figure 4D–F for the respective PIs. Each PI was tested at a constant illumination intensity: I2959, LAP, and EY were illuminated at intensities of 5, 2, or 240 mW/cm^2^, respectively, based on what is most prevalent in the literature.^30,35^ Five different concentrations were tested with each PI. I2959 was tested at a concentration range of 0.01–0.5% w/v, with a maximum *G*′_plateau_ at 0.025% w/v with no significant differences in *G*′_plateau_ between 0.025, 0.05, and 0.1% w/v (*n* ≥ 6, *p* < 0.05; Figure 4A, red). Significant differences in *G*′_plateau_ were evident at very low (0.01% w/v) and to a lesser extent with very high (0.5% w/v) I2959 concentrations. LAP was tested at a concentration range of 0.025–1% w/v, with a maximum *G*′_plateau_ at 0.1% w/v and no significant differences between 0.1 and 0.05% w/v (*n* ≥ 6), yet significant differences were observed for all other LAP concentrations compared to the 0.1% w/v treatment (Figure 4B, blue). EY was tested at a concentration range of 0.02–0.5 mM and had the maximal *G*′_plateau_ at 0.1 mM with no significant difference between 0.1 and 0.2 mM (*n* ≥ 6, *p* > 0.05) and significant differences for all other concentrations compared to 0.1 mM (*n* ≥ 6, *p* < 0.05) (Figure 4F, green). Overall, I2959 and EY reached similar maximum *G*′_plateau_ values of around *G*′ = 270 Pa, whereas LAP reached a maximum *G*′_plateau_ value at about 100 Pa less, representing a 37% reduction (*n* ≥ 6, *p* < 0.0001). Additionally, all PIs exhibited an optimal concentration where a maximum *G*′_plateau_ was reached. Two-way ANOVA confirmed significant differences in the maximum *G*′_plateau_ of the hydrogel as a result of changing the concentration and the PI type, when comparing I2950 or EY to LAP (*n* ≥ 6, *p* < 0.0001), whereas between I2959 and EY, there was no significant difference (*n* ≥ 6, p = 0.95).

**Figure 4:**
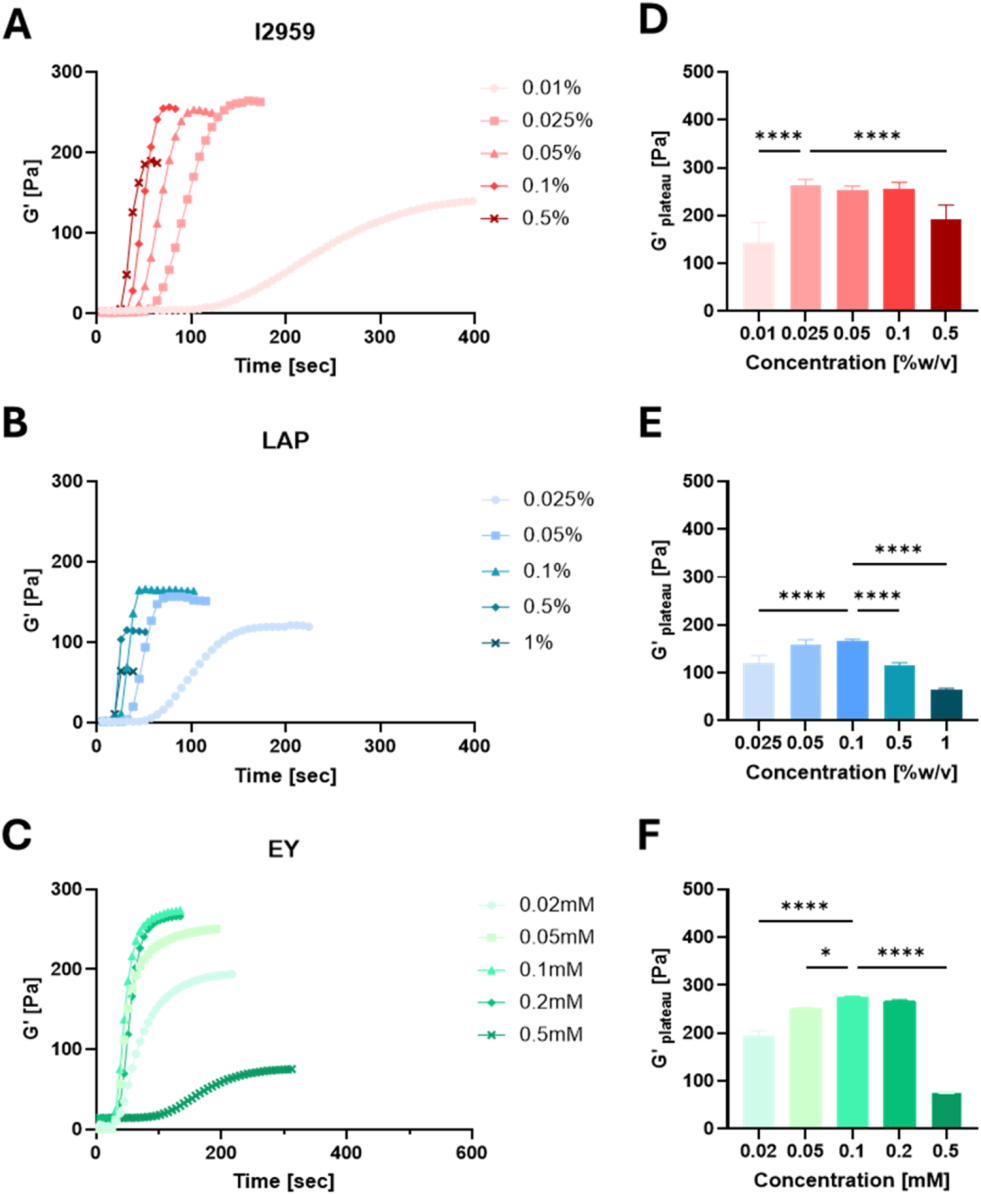
Effect of photoinitiator concentration on the shear storage modulus (*G*′). PF hydrogel (8 mg/mL) samples were cross-linked at room temperature, and irradiation was initiated after 12–15 s. Shear storage modulus *G*′ [Pa] as a function of time for (A) I2959 concentrations 0.01–0.5% w/v, 365 nm, intensity 5 mW/cm^2^; (B) LAP concentrations 0.025–1% w/v, 405 nm, intensity 2 mW/cm^2^; (C) EY concentrations 0.02–0.5 mM, visible light, intensity 240 mW/cm^2^. The plateau shear storage modulus, *G*′_plateau_, for each concentration of I2959 (D), LAP (E), and EY (F). Statistics were calculated using one-way ANOVA—Dunnett’s multiple comparisons test; results are presented in comparison with the treatment exhibiting the highest value (SD, *n* ≥ 6, *P < 0.05, **P < 0.01, ***P < 0.001, and ****P < 0.0001).

#### Evaluating Cross-Linking Kinetics of PIs and Light Intensities

The PF hydrogel cross-linking kinetics are summarized in Figure 5 with respect to PI type, concentration, and irradiation intensity. Each column on the graph displays the cumulative time (values on the left axis) to reach 10, 50, and 90% of the plateau shear storage modulus values (*G*′_plateau_). t_10_ represents the time when photopolymerization begins the formation of the hydrogel, t_50_ represents the photopolymerization time required to achieve half of the maximum cross-linking, and t_90_ represents the photopolymerization time to achieve 90% of maximum cross-linking. Black dots on the graph show the corresponding *G*′_plateau_ values for each treatment (values are on the right axis). When comparing the kinetics data for different concentrations of each PI (Figure 5A), the t_10_ is faster as concentration increases, with I2959 0.01% w/v having the longest t_10_ and longest t_90_ of any other treatment. In addition, the t_10_ and t_50_ shorten as the concentration increases, with all but the EY treatment. With EY, the highest concentration of 0.5 mM results in significant increases in all kinetic parameters. With all three PIs, the *G*′_plateau_ is achieved at the middle range of concentrations tested. Interestingly, photopolymerization with LAP has the fastest overall reaction kinetics with the lowest *G*′_plateau_ values. When comparing the kinetics data for different light intensities (Figure 5B), the data do not follow a uniform dose-dependency with I2959 and LAP, where the intensity has a biphasic effect on kinetics. This biphasic effect is also evident with respective *G*′_plateau_ values of these treatments. For photopolymerization with EY, the increasing light intensity results in faster kinetics and higher *G*′_plateau_ values. The optimal conditions for PF photopolymerization were determined for each PI based on the fastest kinetics and highest *G*′_plateau_ achievable within the tested concentration and intensity range (Table 1). For I2959, this was achieved at 0.1% w/v and 2 mW/cm^2^, for EY this was achieved at 0.1 mM and 240 mW/cm^2^, and for LAP this was achieved at 0.1% w/v and 0.2 mW/cm^2^. Consequently, the *G*′_plateau_ reached with optimal LAP photopolymerization conditions was significantly lower by 30% as compared to *G*′_plateau_ reached with the optimal photopolymerization conditions of the other PIs.

**Figure 5:**
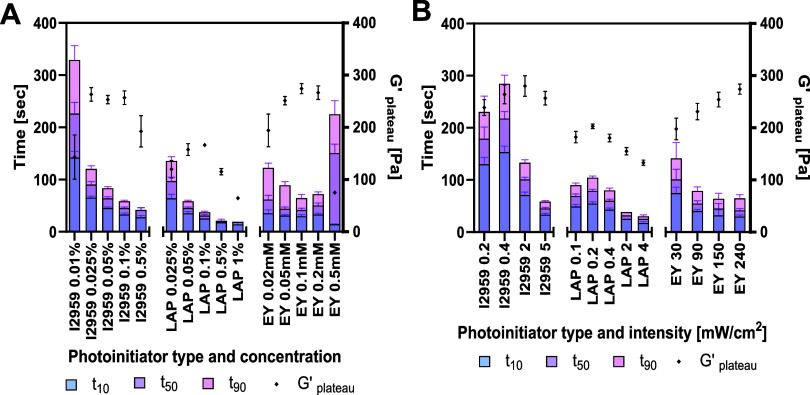
PF hydrogel cross-linking kinetics are affected by photoinitiator type, concentration, and irradiation intensity. Accumulative time to achieve 10, 50, and 90% of the maximum shear storage modulus (*G*′): t_10_, t_50_, and t_90_, respectively. The graphs display the cross-linking time [sec] on the left axis and the resulting plateau shear storage modulus *G*′_plateau_[Pa] on the right axis. (A) Comparison of photoinitiator type and concentration: I2959 concentrations 0.01–0.5% w/v, 365 nm, intensity 5 mW/cm^2^; LAP concentrations 0.025–1% w/v, 405 nm, intensity 2 mW/cm^2^; EY concentrations 0.02–0.5 mM, visible light, intensity 240 mW/cm^2^. (B) Comparison of the photoinitiator type and irradiation intensity: I2959 0.1% w/v, 365 nm, 0.2–5 mW/cm^2^; LAP 0.1% w/v, 405 nm, 0.1–4 mW/cm^2^; EY 0.1 mM, visible light, 30–240 mW/cm^2^. (*n* ≥ 6; results are presented with SD).

**Table 1:** Maximum Value of Shear Storage Modulus (*G*′_plateau_), the Respective t_90_ (Time to Achieve 90% of *G*′_plateau_), and the Conditions Used to Achieve These Values, for All the Hydrogels Tested: PEG-DA, PF, and FibMA

material type	material	PI type	PI conc. [%w/v]	intensity [mW/cm^2^]	*G*′_plateau_ [Pa]	t_90_ [sec]
synthetic	PEG-DA 5% w/v	12959	0.1	2	5132 ± 120	272 ± 21
semisynthetic	PF 8 mg/mL	12959	0.1	2	280 ± 20	137 ± 6
modified protein	FibMA 40 mg/mL	LAP	0.1	0.1	540 ± 115	445 ± 13

### Photopolymerization of Three Different Types of Hydrogels

The time-sweep rheology of photopolymerization and plateau shear storage modulus (*G*′_plateau_) of three different types of hydrogels under different irradiation intensities were evaluated, as depicted in Figure 2B. The data were analyzed in terms of cross-linking kinetics and maximum cross-linking across the different material platforms. Each hydrogel platform reached a different *G*′_plateau_ and responded differently to changes in PI type and light intensity, as further detailed below. For this comparison, only I2959 and LAP were tested because both PIs were found to have optimal photopolymerization conditions (i.e., with a maximum *G*′_plateau_); Eosin Y was not included because we were unable to determine its optimal photopolymerization conditions.

#### Comparing Photopolymerization of the Different Materials

The rheological time-sweep data of the synthetic PEG-DA (Figure 6A) and the semisynthetic PF (Figure 6B) materials are notably different than the modified protein, FibMA (Figure 6C). Two different PIs were used with the three materials, including I2959 (shown in red) and LAP (shown in blue). A constant concentration of 0.1% w/v was used for both PIs, and irradiation intensities of 0.2–5 mW/cm^2^ or 0.1–2 mW/cm^2^ for I2959 or LAP, respectively. The *G*′_plateau_ of PEG-DA, PF, and FibMA as a function of the applied light intensity suggests that optimal irradiation conditions are similar for the synthetic PEG-DA and semisynthetic PF materials but not for the modified biological FibMA material. Optimal irradiation with I2959 (red) yielded a maximum *G*′_plateau_ of 5132 ± 120 Pa at 2 mW/cm^2^. This *G*′_plateau_ is not significantly affected by the change in intensity, with the exception of the lowest intensity tested, 0.2 mW/cm^2^, where *G*′_plateau_ = 4776 ± 194 Pa (*p* < 0.01, *n* > 5). When using LAP (blue), optimal irradiation of PEG-DA yielded a maximum *G*′_plateau_ of 4714 ± 133 Pa at the lowest intensity of 0.1 mW/cm^2^, and a significantly lower *G*′_plateau_ = 4412 ± 171 Pa was measured at the highest intensity of 2 mW/cm^2^.

**Figure 6:**
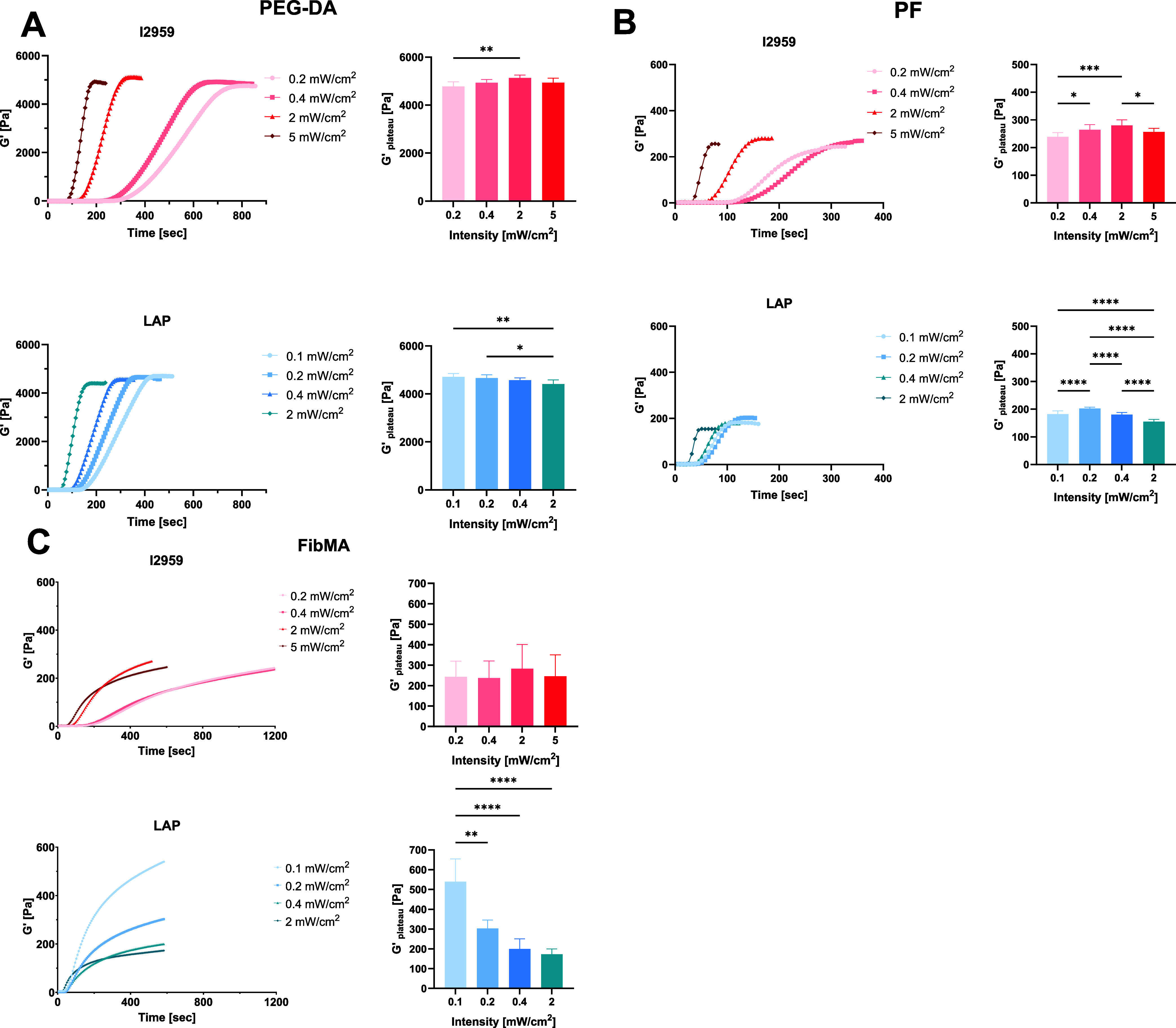
Effect of photoinitiator type (I2959 or LAP) and illumination intensity on shear storage modulus (*G*′) of PEG-DA, PF, and FibMA. The hydrogel samples were cross-linked at room temperature; illumination initiated after 12–15 s. (A) PEG-DA 5% w/v; (B) PF 8 mg/mL; (C) FibMA. Left columns—shear storage modulus (*G*′); Right columns—*G*′_plateau_. Conditions: I2959 0.1% w/v, 365 nm UV light range 0.2–5 mW/cm^2^; LAP 0.1% w/v, 405 nm light range 0.1–2 mW/cm^2^. Statistical analysis was performed using one-way ANOVA—Tukey’s multiple comparisons test (SD, *n* ≥ 6, *P < 0.05, **P < 0.01, ***P < 0.001, ****P < 0.0001).

The photopolymerization of PF followed trends similar to those of the PEG-DA photopolymerization in terms of PI-dependent time-sweep rheometric behavior but not maximal *G*′_plateau_ values. Optimal PF photopolymerization using I2959 (red) achieved a maximal *G*′_plateau_ of 280 ± 20 Pa at an intensity of 2 mW/cm^2^, which was significantly higher when compared to all other intensities except 0.4 mW/cm^2^. The lowest *G*′_plateau_ of 239 ± 15 Pa was obtained with the lowest intensity of 0.2 mW/cm^2^. The optimal photopolymerization conditions for FibMA displayed slightly different trends compared to the other materials, particularly with respect to LAP. Using I2959 (red), the highest *G*′_plateau_ = 283 ± 118 Pa was observed at an intensity of 2 mW/cm^2^ and was not significantly different for any other intensity (*p* > 0.5, *n* = 5). However, when LAP (blue) was used, the highest *G*′_plateau_ = 540 ± 115 Pa was recorded with the lowest intensity of 0.1 mW/cm^2^, with a gradual and significant decrease in *G*′_plateau_ for 0.2–2 mW/cm^2^ and a minimum *G*′_plateau_ value of 173 ± 27 Pa noted at the highest intensity of 2 mW/cm^2^.

#### Comparing Photopolymerization Kinetics of the Different Materials

Evaluation of the time-sweep rheology data provides additional information about the cross-linking kinetics across the three material platforms and PI types. Generally, LAP resulted in faster kinetics as compared to I2959. Moreover, the PF hydrogels exhibited the fastest reaction times, in terms of t_10_, t_50_, and t_90_, followed by PEG-DA and finally FibMA (Figure 7). However, faster kinetics within each material system did not always result in maximum *G*′_plateau_ values, as evidenced by the PEG-DA and PF data, using either I2959 or LAP (Figure 7A,B). This relationship was even more confounded with the photopolymerization of the FibMA, where no clear correlation could be observed regarding the kinetics and *G*′_plateau_ (Figure 7C). Accordingly, the optimal conditions for photopolymerization, based first on the maximum *G*′_plateau_ and second on faster kinetics, were identified for each material system (Table 1). Optimal PEG-DA cross-linking conditions to achieve the highest *G*′_plateau_ while having relatively fast kinetics are with I2959 at 2 mW/cm^2^ intensity (Figure 7A). Consequently, the maximal *G*′_plateau_ value using LAP is significantly lower compared to I2959 (*n* ≥ 6, *p* < 0.0001). Optimal PF cross-linking conditions to achieve the highest *G*′_plateau_ with relatively fast kinetics are with I2959 at 2 mW/cm^2^ intensity (Figure 7B). Optimal FibMA cross-linking conditions to achieve the highest *G*′_plateau_ and relatively fast kinetics are with LAP at 0.1 mW/cm^2^ intensity. In summary, the PEG-DA peaks with 0.1% I2959 at 2 mW/cm^2^ to *G*′ of 5132 Pa after 272 s, the PF peaks with 0.1% I2959 at 2 mW/cm^2^ to *G*′ of 280 Pa after 137 s and the FibMA peaks with 0.1% LAP at 0.1 mW/cm^2^ to *G*′ of 540 Pa after 445 s (Table 1). Interestingly, the variability in *G*′_plateau_ (as measured by standard deviations) is larger for I2959 compared to LAP for the PF and FibMA materials, and larger for FibMA in comparison to PF and to PEG-DA (Figure 7). Correlation analysis using Pearson’s correlation coefficient (*r*) demonstrated that for PEG-DA, PF, or FibMA, there is no good correlation between the kinetics (t_90_) and the corresponding *G*′_plateau_, both for I2959 or LAP (*r* < 0.64).

**Figure 7:**
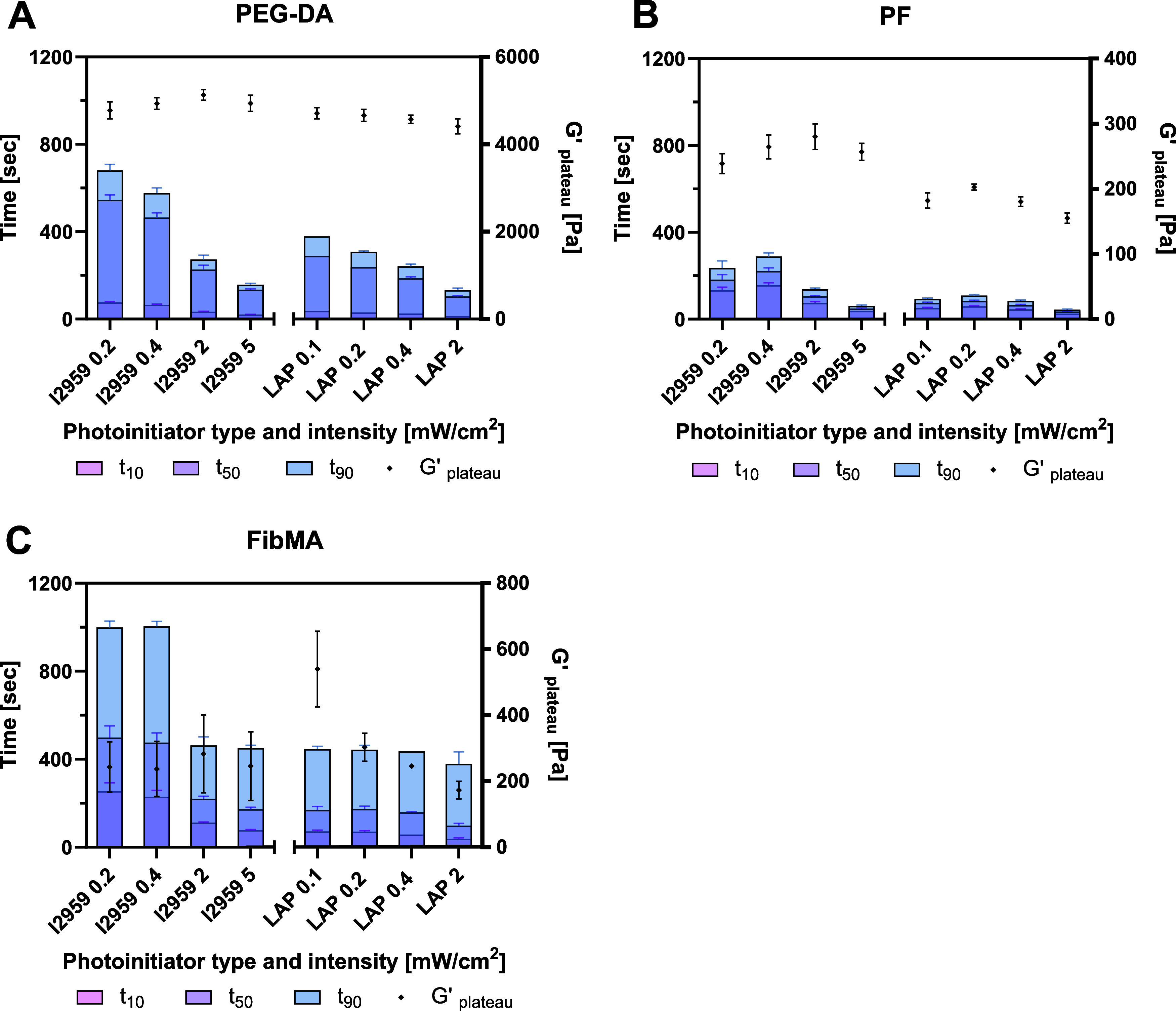
Cross-linking kinetics and maximum shear storage modulus (*G*′_plateau_) of synthetic, semisynthetic, and biologic hydrogels, as a function of photoinitiator type and intensity. t_10_, t_50_, and t_90_ represent the accumulative times to achieve 10, 50, and 90% (respectively) values of the maximum shear storage modulus (*G*′_plateau_). (A) PEG-DA 5% w/v, (B) PF 8 mg/mL, and (C) FibMA 40 mg/mL. I2959:0.1% w/v, 365 nm UV light range 0.2–5 mW/cm^2^; LAP: 0.1% w/v, 405 nm light range 0.1–2 mW/cm^2^. (*n* ≥ 6; results are presented with SD).

### Biocompatibility and Cytotoxicity of the Three Photoinitiators

The cytocompatibility of photopolymerization using different PIs and light intensities was examined by quantifying the viability of NHDF cells (3 × 10^6^ cells/ml) following cell encapsulation in PF hydrogels. The viability was evaluated at maximal concentration and irradiation conditions for all PIs, with the exception of LAP, which was evaluated at 0.025, 0.1, or 1% w/v and illuminated at 4 mW/cm^2^ (depicted in Figure 2C). Both I2959 and EY had >90% viability using the maximal concentration and irradiation conditions, with no statistically significant difference between them (*p* < 0.01, *n* > 5; Figure 8A). The LAP, when applied at the maximal concentration and irradiation conditions (i.e., 1% w/v and 4 mW/cm^2^), had ∼80% viability and was statistically lower than the other two PIs at their highest concentrations and intensities (*p* < 0.05, *n* = 5; Figure 7B). Further testing with lower concentrations of LAP, including 0.025% and 0.1% w/v, proved effective in increasing the viability to >90% with statistical significance compared to the highest concentration of LAP (Figure 8B). A two-way ANOVA comparing 0.1% w/v LAP results with the other PIs indicated no significant difference in and between groups, with a viability of above 90% (*p* > 0.05, *n* ≥ 6). Cell viability was further confirmed using the Calcein/Ethidium Live/Dead assay, which stained the live cells in green and dead cells in red (Figure 8C). Live/Dead staining indicated that cells in all tested formulations, which were initially round (after 2 h, upper row in Figure 8C), began to spread and extend lamellipodia within the hydrogels after 24 h (Figure 8C, lower row).

**Figure 8:**
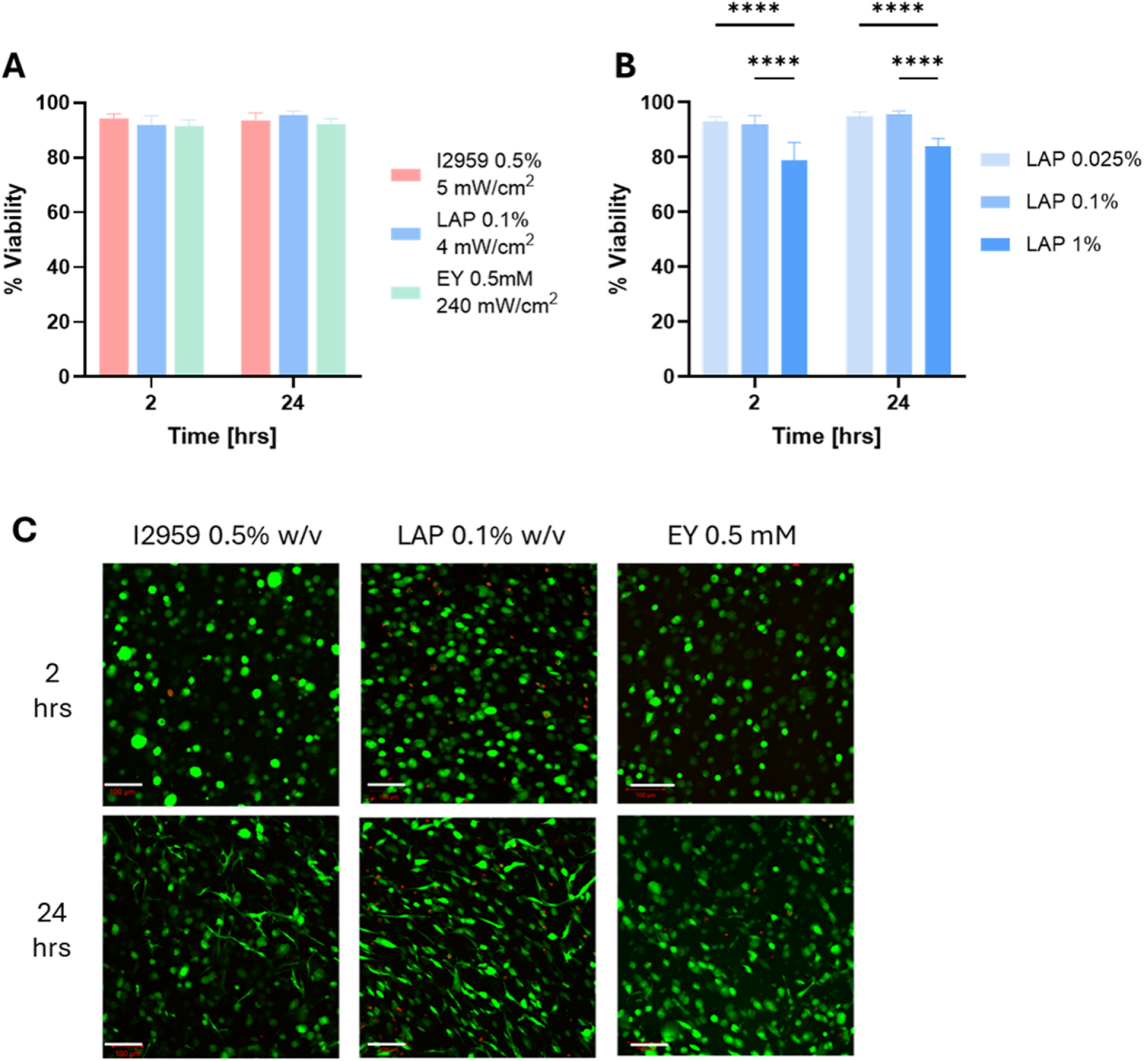
Cell viability and cytotoxicity associated with the different PIs tested in PF hydrogels. (A) Cell viability of neonatal human dermal fibroblasts (NHDF) following photopolymerization in PF hydrogels: shown are 2 and 24 h of 3D culture. Hydrogel plugs illuminated for 3 min at the maximum light intensity of each PI, then placed in a 24-well plate with medium for 2–24 h. Red—I2959 0.5% w/v, 365 nm, illuminated at 5 mW/cm^2^; blue—LAP 0.1% w/v, 405 nm, illuminated at 4 mW/cm^2^; and green—EY 0.5 mM, visible light, illuminated at 240 mW/cm^2^. No significance between groups, *n* ≥ 7. (B) Cell viability of NHDF at different LAP concentrations (0.01, 0.1, and 1% w/v) at a maximum irradiation of 4 mW/cm^2^ (*n* ≥ 4, ****P < 0.0001). (C) Fluorescence imaging of NHDF in 3D culture within PF hydrogels following photopolymerization after 2 h (upper) and 24 h (lower). The NHDF cells (3 × 10^6^ cells/ml) were encapsulated in 8 mg/mL PF hydrogels and illuminated for 3 min with maximum light intensity of each PI to form 3D plugs. Calcein staining (green) represents live cells, and ethidium staining (red) represents dead cells. Scale bar: 100 μm. Left—I2959 0.5% w/v, 365 nm, illuminated at 5 mW/cm^2^; middle—LAP 0.1% w/v, 405 nm, illuminated at 4 mW/cm^2^; and right—EY 0.5 mM, visible light, illuminated at 240 mW/cm^2^. (Statistics were calculated using one-way ANOVA—Tukey’s multiple comparisons test. Results are presented with SD, *p* > 0.05, *n* ≥ 5).

## Discussion

Light-activated polymerization, or photopolymerization, is commonly applied to rapidly cross-linking hydrogels with minimal cytotoxicity.^41,42,51^ Although photopolymerization requires an optical window due to a lack of light penetration into deep tissues, in applications where an optical window is present, this technique has several advantages, particularly in biomedical applications and 3D bioprinting. The cross-linking mechanism of photopolymerization is either by radical polymerization or by cationic polymerization. Cationic photopolymerization is applied in industrial applications to cross-link polymeric materials but cannot be used for hydrogels in biomedical applications. This is because cationic photopolymerization is highly sensitive to moisture and the presence of water slows down or completely inhibits the reaction,^52^ and cationic initiators generate strong protonic acids that are detrimental to cells.^31^ Alternatively, radical photopolymerization is a chain reaction, whereby a photoinitiator (PI) forms radicals upon irradiation to start the cross-linking, followed by propagation (chain growth) and termination^53^ (Figure 1). Photochemistry works well in aqueous medium, provided that the PI is sufficiently water-soluble.^54^ Spatial control of photochemistry is easily attained using conventional masking techniques.^29,30^ We and others have shown that radical photopolymerization allows hydrogel cross-linking in the presence of cells with minimal cytotoxicity.^28,38,55^

The reaction kinetics of photopolymerization are relatively fast, with hydrogels forming within seconds to minutes.^56^ Faster reaction kinetics are important in applications that require in situ polymerization, specifically when the gelation can be altered by dilution of the precursors with interstitial fluids.^31^ Moreover, photopolymerization of hydrogels enables preparation and placement of a homogeneous precursor solution in advance, followed by a temporally controlled cross-linking reaction upon activation by irradiation. Unfortunately, it is difficult to predict radical polymerization reaction kinetics in a dilute polymer solution, which not only affects the reaction duration but can also have an impact on important hydrogel properties associated with cross-linking density that include the mesh size (porosity), transport and diffusion properties, mechanical strength, modulus, and viscoelasticity.^42,57^ Ultimately, understanding the chain reaction of hydrogel precursor cross-linking can alleviate the unpredictability of applying photopolymerization to new hydrogel formulations.

The most important factor that affects hydrogel photopolymerization is the polymer system, which is characterized by the concentration of the polymers in solution, the degree of functionalization, the thickness of the prepolymerized construct, the influence of additives in the polymer solution prior to cross-linking, and the viscosity of the precursor polymer solution. The three hydrogel platforms tested in this study exhibit different *G*′_plateau_ values because of differences in their monomer concentrations and degree of functionalization. However, it is important to note that all hydrogels in this study, particularly the semisynthetic hydrogels, reached their expected *G*′_plateau_ values as determined in previously published reports.^16,23^

Another critical factor that affects hydrogel photopolymerization cross-linking is the photoinitiation system. PI type, concentration, light source, and irradiation conditions can alter the efficiency of the photopolymerization cross-linking reaction. A more complete understanding of the interactions between the polymer system and the photopolymerization system can help to reduce any unwanted variability in the photopolymerized hydrogel properties. Importantly, any modifications to the photopolymerization protocol for a biomedical hydrogel must be made with careful consideration of the consequences on cytocompatibility. Accordingly, we set out to investigate three commonly used PIs (I2959, LAP, and EY) in terms of their cytocompatibility and effectiveness in cross-linking different types of biomedical hydrogels. It is useful to note that there are many other photoinitiators used for photopolymerization, but these three PIs are broadly applied for biomedical polymers, including hydrogels, because of their water solubility^30,58^ and minimal cytotoxicity.^29,30,37,42^

## PI Influence on Hydrogels Cross-Linking

Photoinitiators for biomedical applications are divided into two main types. Type 1 photoinitiators include photoinitiators that form free radicals in a single-step process. This group includes ketones, peroxides, iminosulphones, and peresters.^31^ In the case of type 2 photoinitiators, the free radicals are formed in a two-step process, where the excited initiator molecule reacts with an appropriate co-initiator to produce the radicals^59^ (Figure 1A). I2959 is one of the most popular type 1 photoinitiators for hydrogel cross-linking, used for the photopolymerization of materials such as PEG-DA,^60^ PF,^37,61^ GelMA,^62^ FibMA,^23^ and others, as well as for encapsulation of cells and drugs for various biomedical applications.^42^ It contains a ketone group as a functional group and has a relatively narrow absorption range only in the UV spectrum, with maximum absorption at 276, which is highly toxic for cells.^63^ Because of this toxicity, I2959 is irradiated above 350 nm, or precisely at 365 nm, depending on the light source, and its molar extinction coefficient at 365 is ε = 4 M^–1^cm^–1^.^28^ When I2959 is irradiated, it is cleaved into two radicals, alkyl and benzoyl; both can initiate the polymerization reaction.^46^ Another type 1 photoinitiator is LAP. LAP is a phosphine derivative, with high water solubility and good spectroscopic properties^28,64^ (Figure 1A). It is a wildly used photoinitiator in recent years for obtaining hydrogels for biomedical applications, including GelMA,^65^ PEG-DA,^66^ and other monomers.^42^ LAP has maximum absorption at approximately 375 nm. LAP’s molar extinction coefficient at 365 nm is ε = 218 M^–1^cm^–1^. Additionally, LAP absorbs in the visible region from 400 to 420 nm, with a molar extinction coefficient at 405 nm of approximately ε = 20 M^–1^cm^–1^.^28^ EY is a type 2 photoinitiator, which needs a second molecule (co-initiator) to initiate the polymerization reaction^67^ (Figure 1A). Following irradiation, EY is excited to a triplet state and becomes an acceptor of the electron given by the co-initiator (e.g., amine). The result of this process is the formation of EY’s radical anion and the co-initiator radical cation. Then, there is a proton transfer, and two neutral radicals are formed.^48^ EY has good water solubility and spectroscopic properties (maximum absorbance at 528 nm) and hence is suitable for use with light sources in the visible range. EY is used for targeted drug delivery, surface photopolymerization for encapsulation of living cells, and obtaining hydrogels for biomedical purposes.^42^

The first set of our results show that the choice of PI can significantly affect hydrogel cross-linking by measuring the rheological properties of the precursor solution during photochemistry. Rheological measurements of shear storage modulus (*G*′) are frequently applied to evaluate the mechanical properties of biomedical hydrogels and are associated with cross-linking density and mesh size.^2,68^ Here, we focused on the semisynthetic PF hydrogel and compared three PIs, in three distinct excitation wavelengths (Figure 1C), in a range of concentrations and irradiation intensities based on previously published parameters.^1,28,31,35,37,38,51,69^ The mechanical properties of the final PF hydrogel was evaluated in terms of *G*′_plateau_ ^21^ and the kinetics of the cross-linking reaction (as measured by time to reach *G*′_plateau_).^21^ We observed that I2959 and EY reached similar *G*′_plateau_ values of around 280 Pa, whereas LAP (at 405 nm) was significantly weaker by 30% with a *G*′_plateau_ of around 200 Pa. This may be related to the molar extinction coefficient of LAP (∼20 M–1 cm–1), which is higher compared to I2959 (∼4 M^–1^ cm^–1^). The absorbance of LAP at 405 nm is higher, so more radicals are formed, which can lead to inner loop formations or terminations that do not contribute to the network cross-linking and can lead to lower shear modulus.^33^

In terms of the concentrations and irradiation intensities of the PIs, there was an optimal formulation for I2959 and LAP that achieves the maximum hydrogel shear storage modulus (optimal parameters for EY were not determined due to light source limitations, as specified below). Consequently, a higher shear storage modulus represents a more efficient cross-linking reaction, resulting in a higher cross-linking density of the hydrogel. We presume that at the lower concentrations of PI, fewer free radicals are formed, whereas at higher concentrations, an abundance of free radicals can increase the number of terminations and hence fewer bonds between the hydrogel molecules or shorter polymer chains, both causing lower *G*′. When evaluating the effect of light intensity on cross-linking kinetics, I2959 was most affected by irradiation time; at lower intensities, it can take twice as long to achieve full cross-linking, compared to higher intensities. The reason for that can be the small number of I2959 molecules excited at lower intensities. In the case of I2959 and LAP, there is a decrease in *G*′_plateau_ for the higher intensities, possibly due to many radicals formed, which can lead to more terminations.^33^ In the case of EY, higher light intensities could have further increased (or decreased) the *G*′_plateau_; our light source was limited to 240 mW/cm^–2^, and we were not able to identify optimal EY irradiation parameters beyond this value. Unlike I2959 and LAP, the highest concentration of EY resulted in significantly slower reaction kinetics, possibly due to light attenuation effects.^70^ Others have shown that high concentrations of EY decrease the intensity of visible light that can penetrate the material.^49,70^ These results show that different PI types, their concentrations, and light intensities can cross-link the same material in different kinetics that can affect the mechanical properties of the PF hydrogels.

## PI Influence on Different Types of Biomedical Hydrogels

Next, we assessed how two different PIs (I2959 and LAP) influenced cross-linking across three different hydrogel platforms. The three types of biomedical hydrogels were a synthetic PEG-DA (10 kDa 50 mg/mL), a semisynthetic PF (8 mg/mL), and a modified protein, FibMA (40 mg/mL). The PI concentrations were held constant (0.1% w/v), and the irradiation intensities varied between 0.2 and 5 mW/cm^2^ for I2959 and 0.1 and 2 mW/cm^2^ for LAP. We observed that the synthetic PEG-DA had optimal cross-linking conditions using I2959 at 2 mW/cm^2^, without significant differences from most other intensities. This result is consistent with previously published studies.^37^ When comparing to LAP, the maximal values were approximately 8% smaller compared to I2959 (*n* > 6, *P* < 0.0001). For both PIs, the time to achieve 90% of the plateau shear storage modulus decreases with an increase in intensity, with small or nonsignificant differences in the final *G*′_plateau_. These results indicate that kinetics does not affect the maximum storage modulus with PEG-DA hydrogels, but the light intensity does significantly affect the photopolymerization time of PEG-DA because the time to achieve *G*′_plateau_ decreases with the increase in intensity (*n* ≥ 6, *P* < 0.0001). It is also apparent that I2959 may be better than LAP for achieving higher strength PEG-DA hydrogels.

For the semisynthetic PF, we found that the optimal cross-linking conditions to achieve the highest shear storage modulus were using I2959 at 2 mW/cm^2^ (same as with PEG-DA). For LAP, 0.2 mW/cm^2^ gave the highest *G*′_plateau_. The kinetics and *G*′_plateau_ values showed a local maximum for both I2959 or LAP, suggesting that the optimal time and light intensity to achieve the highest cross-linking density were reached (Figure 7). As mentioned earlier, the *G*′_plateau_ of PF with I2959 was approximately 30% higher in comparison to LAP. The FibMA hydrogel *G*′_plateau_ values when using I2959 were similar for all intensities, with relatively large standard deviations evident. The kinetics to achieve the highest *G*′ were half the time with higher intensities (2–5 mW/cm^2^), compared to the lower intensities (0.2–0.4 mW/cm^2^). When using LAP, the kinetics were similar regardless of the intensity. There was also a much larger difference between the *G*′_plateau_ values of FibMA when using different LAP intensities; there was a gradual decrease in *G*′_plateau_ with the increase in intensities (Figure 7). The highest *G*′_plateau_ was obtained for the lowest intensity of 0.1 mW/cm^2^ and was significantly higher compared to all other values. Because FibMA is a methacrylated protein, it behaves more like a native protein with high variability as compared to the synthetic PEG-DA and the semisynthetic PF. This may explain the high standard deviations in part of the FibMA results. We speculate that the high variability may be due in part to FibMA’s macromolecular motility in solution, which can affect photochemistry reactions.^71^ The FibMA can only cross-link at a minimum concentration of 40 mg/mL or higher,^23^ suggesting that the protein dilution plays a role in photochemistry, and mobility hinders methacrylic groups on fibrinogen chains from bridging the distance between them to bind to each other during the reaction. This also explains why the kinetics are significantly longer for FibMA compared to PEG-DA and PF. Consequently, addition of a cross-linker such as PEG-DA may be used at lower concentrations of FibMA to bridge the methacrylic groups more readily.^23^ Macromolecular mobility can also be rate-limiting during photochemistry of PF hydrogels, although to a lesser extent than FibMA because the PEG bound to the fibrinogen acts like a chain extended when facilitating acrylate cross-linking.^72^ Our earlier work reported that the minimum concentration of PF required for radical photopolymerization is about 6 mg/mL when the PF is made from 10 kDa PEG-DA.^72^

## Cell Viability and Cytotoxicity Following Photopolymerization Using Different PIs

The cytotoxicity of I2959, EY, and LAP was evaluated by NHDF cell viability after photopolymerization in PF hydrogels. We chose to test viability first on the most extreme conditions, namely, the highest concentrations and highest intensities for each respective PI. The rationale of this approach is that if the highest concentration and intensity are nontoxic (i.e., > 90% viability), then the lesser concentrations and intensities would presumably also be nontoxic. High I2959 (0.5% w/v) and EY (0.5 mM) had >90% viability with their respective highest intensities. With the LAP at the highest concentration of 1% w/v and the highest intensity, cell viability was <80% after 2 h and significantly lower than the other two PIs (*p* < 0.0001, *n* = 9). This prompted further testing to identify a lower concentration that produced >90% viability at the highest intensity. This was determined experimentally to be 0.1%, as indicated in Figure 8B. When comparing viability results of the three PIs, the highest concentration of LAP was 34 nM, nearly twice as much as the highest concentration of I2959 (22 mM) and 68 times higher than EY (0.5 mM). This may explain why LAP appears to be slightly more cytotoxic to cells with respect to EY or I2959 at the highest concentrations tested. The decreased LAP concentrations of 0.025% and 0.1% w/v (1 mM and 3.4 mM, respectively) resulted in greater than 90% cell viability and were statistically similar to I2959 and EY at the highest concentrations tested. Light intensity was less consequential regarding viability because even in the highest light intensities applied, cell viability remained high for all PIs (>90%, *p* < 0.0001, *n* ≥ 4). It is important to note that the NHDF cells used are known to be resilient to photochemistry and specifically to long-wave UV PIs. As such, these results may not have widespread implications for other, more sensitive cell types. Indeed, there has always been a preference toward irradiation in the visible light range when working in the presence of cells and tissues, due to possible cell damage following long-wave UV exposure,^73^ and the fact that some cell types are more sensitive than others and cannot survive UV irradiation.^28,38,58^ In such cases, the possibility of using LAP or EY instead of I2959 should be considered. EY has a long track record of cytocompatibility when working with cell-laden hydrogel photopolymerization but requires a higher intensity light source to facilitate the reaction.^74^ LAP works at the lower end of the visible light spectrum and thus requires less light intensity to initiate the reaction. However, less is known about LAP’s cytocompatibility with different cell systems because it is a relatively new PI in biomedical photopolymerization.^28,30,38^

The cytotoxicity of photochemistry is a complex topic that includes toxicity from the PI, radical formation, and possibly exposure to UV light. It is well established that cells subjected to low wavelength UV light can cause DNA damage and cell death. It is also well-known that free radicals formed during the photopolymerization reaction can cause cell death, depending on their amount and their rate of formation. When working with cell types that are sensitive to long-wave UV light, LAP is a good alternative PI, but radial formation may still cause cell death. Here, we found that LAP at the highest concentration causes cell death at the highest light exposure tested, although we did not determine if toxicity was caused by the abundance of LAP or the abundance of free radicals during photochemistry. Others have reported reduced cell viability after photochemistry with similarly high LAP concentrations using GelMA hydrogels and human primary renal proximal tubule epithelial cells (hRPTECs),^75^ purportedly caused by an abundance of LAP. Importantly, we also found a 30% reduction in the mechanical properties of the hydrogel when using LAP (even under optimized conditions). Taken together, our results indicate that cytocompatibility must be factored with other considerations of photochemistry, namely, the reaction conditions leading to the maximum cross-linking of the hydrogel network. We observed that the photo-cross-linking process depends on the PI type and light intensity applied and that this is different for each material. Therefore, one cannot assume the effect on a specific hydrogel based on what is known in the literature for different type of hydrogel, and a specific examination of the PI and conditions should be applied for each material to achieve the optimal desired characteristics. In this context, we chose to examine our cytotoxicity with a semisynthetic PF that has been used with various cell types.^17,37,61,76^

There are a few limitations in our study, mainly associated with the challenge of comparing photochemistry across different hydrogel platforms and PI types. For example, it is expected that different monomers, polymer concentrations, and functional groups will exhibit different reactions, resulting in differing modulus values. These differences complicate our modulus comparisons across different material platforms. To overcome these differences, we sought a local maximum *G*′_plateau_ that would be associated with each photochemical reaction treatment condition (i.e., PI concentration or light intensity). If we assume that the cross-linking reaction for each treatment condition and each platform reaches its maximum cross-linking at this inflection point, then we can assign an optimal cross-linking per treatment condition for each respective monomer solution. Comparison of the locally optimized cross-linking is one way to normalize between the different functional groups and monomer compositions in our study. Unfortunately, we were unable to achieve a local maximum for the EY with our experimental system, which is a limitation of the study. Another limitation of this study relates to the cytocompatibility results using NHDFs, which tend to be more tolerant to radical polymerization when compared to other more specificized cell types such as aortic smooth muscle cells^77^ or mesenchymal stem cells.^40^ In our study, we use NHDFs as a baseline to compare with similar studies performed in previous studies.^27,29,33,37^ However, future studies would be required using other cell types, including mesenchymal stem cells, to provide a more complete understanding of the cytocompatibility of PIs as related to the cross-linking reaction.

## Conclusions

Photopolymerization is a good method to form hydrogels, with or without encapsulated cells, if one can ensure proper cross-linking while maintaining high cell viability. The goal of this research was to systematically compare the photopolymerization process taking place with three commonly used photoinitiators, including I2959, LAP, and EY, to better understand the influence they have on hydrogel mechanical properties and cell viability. Furthermore, we aimed to assess how the material choice can impact the PI performance in terms of efficiency of the cross-linking reaction. We conclude that when choosing a biomedical hydrogel, it is of high importance to perform rheological measurements with the different PIs to find their best working conditions (i.e., concentration and light intensity) for maximum cross-linking efficiency. Regarding LAP’s cross-linking efficiency, we conclude that it had no significant advantage over I2959 or EY and produced consistently weaker gels, irrespective of the material choice. We hypothesize that LAP produced a suboptimal photochemical reaction, causing poor cross-linking and reduced mechanical properties. All PIs tested have good viability when used in moderate concentrations. Importantly, we conclude that optimization of PI cross-linking across different material platforms is necessary because mechanical properties will be affected by the choice of PI. Moreover, PI type, concentration, and light intensity affect the cross-linking kinetic profile (i.e., the sol–gel transition kinetics). Taken together, we conclude that without careful consideration and investigation of the PI choice, one can inadvertently alter the chemical and physical properties of the final hydrogel.

## Supplementary Material



## Notes

Additional experimental results, including NMR characterization of PEG-DA and conversion tables for calculating PI concentrations (PDF)
