# Supplementary material for: Evaluating Cross-Linking Efficiency and Cytocompatibility of Three Commonly Used Photoinitiators across Different Cell-Compatible Hydrogel Platforms
Source: Biomacromolecules. 2025 Sep 22;26(10):6817–33. doi: 10.1021/acs.biomac.5c01142 (PMC12522141; doi:10.1021/acs.biomac.5c01142)

Evaluating crosslinking efficiency and cytocompatibility of three commonly used photoinitiators across different cell-compatible hydrogel platforms

Aya Gavish Moscovitz[1], Haneen Simaan Yameen[1,2], Orit Bar-Am[1] and Dror Seliktar[1]

[1] The Faculty of Biomedical Engineering, Technion-Israel Institute of Technology, Haifa, Israel

[2] The Interdisciplinary Program in Biotechnology, Technion–Israel Institute of Technology, Haifa Israel

Supplementary Data

Photoinitiator Conversion Tables

Eosin Y Disodium Salt: mM to % w/v

mM	% w/v
0.02	0.001384
0.05	0.00346
0.1	0.00692
0.2	0.01384
0.5	0.0346
Formula	%w/v = mM × 0.0692

Conversion formula: % w/v = mM × 0.0692 (MW = 692 g/mol)

LAP: % w/v to mM

% w/v	mM
0.025	0.8497
0.05	1.6995
0.1	3.3989
0.5	16.9947
1.0	33.9893
Formula	mM = (%w/v × 10^4) / 294.21

Conversion formula: mM = (% w/v × 10^4) / 294.21 (MW = 294.21 g/mol)

Irgacure 2959: % w/v to mM

% w/v	mM
0.01	0.4459
0.025	1.1148
0.05	2.2296
0.1	4.4591
0.5	22.2955
Formula	mM = (%w/v × 10^4) / 224.26

Conversion formula: mM = (% w/v × 10^4) / 224.26 (MW = 224.26 g/mol)

1H-NMR of PEG-DA (10 kDa)

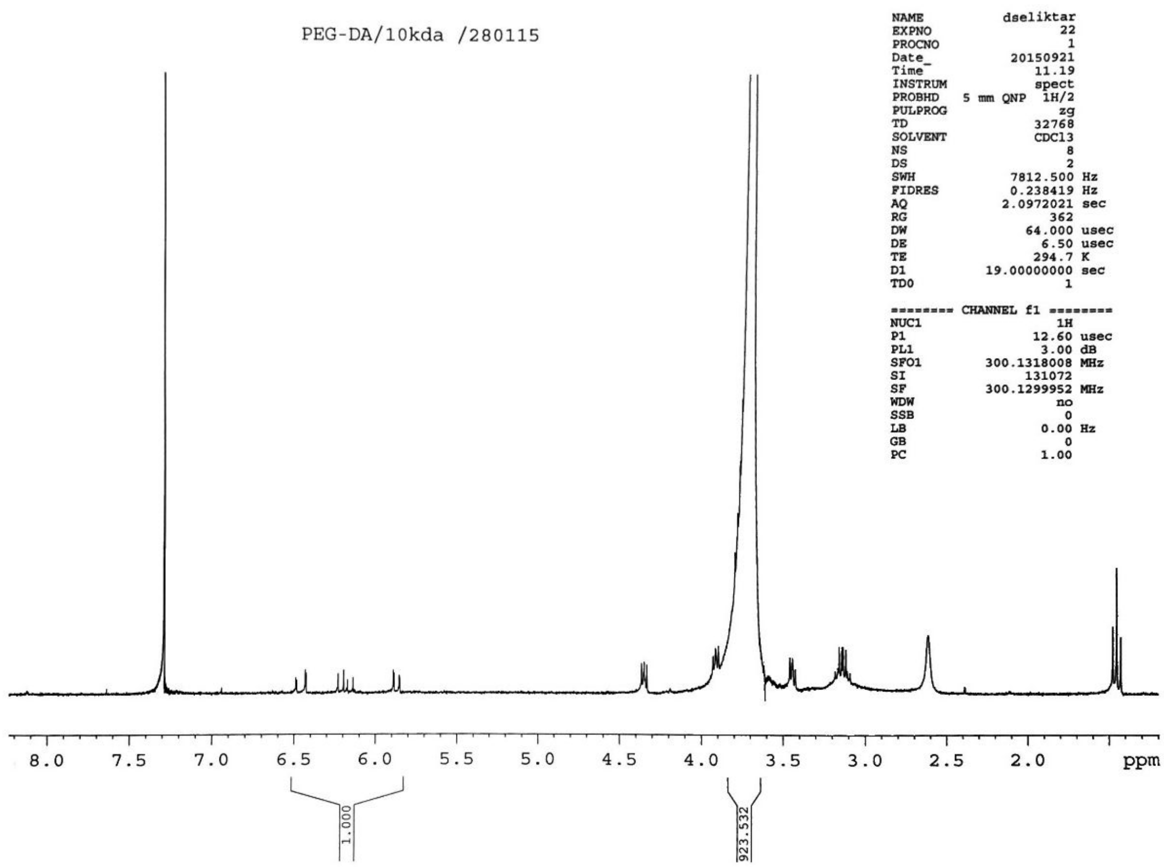

Supplement: Supplementary file 1 [file bm5c01142_si_001.pdf]
